# “Watch and Wait” Strategy for Multicystic Dysplastic Kidney (MCDK): Status Survey of Perceptions, Attitudes, and Treatment Selection in Chinese Pediatric Urologists and Pediatric Surgeons

**DOI:** 10.3389/fped.2020.00423

**Published:** 2020-07-28

**Authors:** Qi Wang, Zhengzhou Shi, Dapeng Jiang

**Affiliations:** Department of Urology, Shanghai Children's Medical Center, Shanghai Jiao Tong University School of Medicine, Shanghai, China

**Keywords:** China, prenatal diagnosis, vesicoureteral reflux, voiding cystourethrogram, survey

## Abstract

To investigate the perceptions, attitudes, and treatment selection of Chinese pediatric urologists and pediatric surgeons regarding a “watch and wait” strategy for multicystic dysplastic kidney (MCDK). We used a cross-sectional survey in this study. We sent the questionnaire to pediatric urologists and pediatric surgeons to capture their views via the “Questionnaire Star” online survey platform between November and December 2019. The questionnaire contained the basic information and surgical experiences of the respondent, respondents' awareness regarding the counseling of prenatally-diagnosed MCDK and the treatment of MCDK, and respondents' knowledge regarding the imaging modalities, frequency, and duration of follow-up. Of the 200 questionnaires we sent, we received 151 responses. Of those 151 complete responses, most respondents were women (*n* = 104, 68.9%), pediatric urologists (*n* = 78, 51.6%), and practicing with at least 5 years of surgical experience (*n* = 112, 74.2%); 11.9% reported >20 years' experience. Eighty-two surgeons (54.3%) provided positive counseling for prenatally-diagnosed MCDK. Ninety-nine surgeons (65.6%) advocated conservative management for MCDK, and only 14.8% of respondents suggested limiting the use of radiographic evaluation for MCDK. Surgeons working in academic teaching facilities and those from East China were more likely to select a “watch and wait” strategy. Chinese pediatric urologists and pediatric surgeons have inadequate knowledge of the “watch and wait” strategy for MCDK. An expert consensus on the strategy of “watch and wait” for MCDK in China is urgently needed to promote the application of this non-surgical treatment mode in clinical practice. A larger sample size is required to fully identify the current opinion of Chinese pediatric urologists and pediatric surgeons regarding the management of MCDK.

## Introduction

Multicystic dysplastic kidney (MCDK) is a congenital urinary tract anomaly, and is the most common form of cystic kidney disease in children. With the popularization of prenatal ultrasound screening and improvements in ultrasound technology, the diagnosis of asymptomatic renal anomalies is increasing (1, 2). In patients with MCDK, the embryonic structure and the development of the renal cortex in the involved kidney(s) are abnormal, and are replaced by multiple non-communicating cysts of varying sizes. Several studies have shown that MCDK has the tendency to involute (3–6), and the involution rate increases with age, which explains why MCDK is rarely found in adults. The rate of hypertension in children with MCDK is lower than in the general population (7). Another potential concern associated with MCDK is malignancy; fortunately, children with MCDK have a very low risk of malignancy. Moreover, the grade of vesicoureteral reflux associated with MCDK is often low (grade I–II), and in the majority of patients, this resolves spontaneously (8–10). Considering the low risk of hypertension, tumor formation, and urinary tract infection (which is similar to the risk in the general population), conservative non-surgical management is a suitable treatment strategy for MCDK. Therefore, many scholars suggest ways to support clinicians to dynamically observe and manage these patients.

A “watch and wait” strategy is advocated according to recent natural history studies of MCDK (2). However, in some institutions, routine early surgical removal of these kidneys was advocated previously because of concerns about the potential for hypertension and malignancy (11, 12). The appropriate management strategy for this anomaly is debated, and strategies are inconsistent within and between institutions. The debate surrounding MCDK involves information regarding the management options and prognosis, which are provided by pediatric urologists and pediatric surgeons after a prenatal diagnosis of MCDK, and whether routine voiding cystourethrograms (VCUG) to evaluate vesicoureteral reflux are necessary, whether renal scans are necessary, whether nephrectomy is recommended as part of the management algorithm of MCDK, and finally, the appropriate number and frequency of renal ultrasound evaluations.

Both pediatric urologists and pediatric surgeons assess and manage children with MCDK in China. These surgeons may have different perceptions owing to their different experiences and training background. This study was conducted to investigate the awareness, attitudes, and practice patterns of Chinese pediatric urologists and pediatric surgeons regarding the treatment of MCDK. We performed a survey of these surgeons who perform pediatric urological surgery to clarify these items.

## Methods

### Study Design and Participants

This study was performed in accordance with the principles of the Declaration of Helsinki. This study was approved by the Shanghai Children's Medical Center Research Ethics Committee (SCMCIRB–W2020005). We used a 22-question cross-sectional survey in this study, and the survey was designed and reviewed by three experienced pediatric urologists who have a particular interest in MCDK. The survey included seven questions to assess the participants' demographic characteristics, one question to assess their surgical experience regarding pediatric urological diseases, and three questions to assess respondents' awareness regarding the recommendations and counseling for prenatally-diagnosed MCDK. Eight questions were developed to survey the practices and attitudes of responding pediatric urologists and pediatric surgeons regarding the treatment of MCDK, and one question assessed respondents' knowledge regarding the natural history of MCDK. The penultimate question in the survey concerned the choice of imaging modalities.

There are 2,500 pediatric urologists/pediatric surgeons work in China overall. Pediatric urologists and pediatric surgeons were eligible to participate in this survey if they treated or followed patients with MCDK. We sent the questionnaire to pediatric urologists and pediatric surgeons via the “Questionnaire Star” online survey platform between November and December 2019. To improve survey response rates, we called the heads of pediatric surgery or pediatric urology departments in different regions to remind their staff to complete the questionnaire, and increased awareness of the ongoing survey by advertising during professional meetings. Data were collected from November 2019 to January 2020.

### Statistical Analysis

Data are reported as mean ± SD for continuous data and percentage for categorical variables. Chi-squared tests or Fisher's exact test was used to compare differences in responses. Comparison of respondents' attitudes to MCDK based on years of experience used Cochran-Armitage trend test. *Post-hoc* test was used to describe and compare multiple comparisons of groups. A 2-sided *p*-value of <0.05 was considered statistically significant. All statistical analyses were performed by using SPSS 23.0 software.

## Results

### Demographic Characteristics of the Participants

During the study period (3 months), 151/200 surveys were completed (75.5% response), and all 151 surveys had 100% of the items completed. The first analysis step was to provide a descriptive overview of the sample. The demographics of the respondents are displayed in Table 1. The mean age of the respondents was 41.7 ± 10 years, and the majority of the respondents were female (*n* = 104, 68.9%). Some surgeons (47.1%) were employed at academic teaching hospitals and approximately half (51.6%) practiced in a department of pediatric urology. The majority of respondents were from East China (*n* = 70, 46.4%). The education level of most participants was the master's level and above (*n* = 115, 76.2%), but others had bachelor's degrees (*n*=36, 23.8%). Most respondents (*n* = 112, 74.2%) reported having at least 5 years of surgical experience.

**Table 1:** Demographics of 151 respondents.

**Characteristic**	**Participants [No. (%)]**
**Age (years)**	
Up to 35	29 (19.2)
36–45	77 (51)
46–55	35 (23.2)
More than 56	10 (6.6)
**Sex**	
Male	47 (31.1)
Female	104 (68.9)
**Years of experience**	
<5	39 (25.8)
5–10	50 (33.2)
11–20	44 (29.1)
>20	18 (11.9)
**Medical specialty**	
Pediatric urology	78 (51.6)
Pediatric surgery	73 (48.4)
**Geographic location**	
Northeast	30 (19.9)
West China	28 (18.5)
East China	70 (46.4)
Central China	23 (15.2)
**Education level**	
Bachelor	36 (23.8)
Master and above	115 (76.2)
**Practice setting**	
Academic teaching facility	71 (47.1)
Private practice	3 (2)
local or community hospitals	10 (6.6)
Public/Government hospital	67 (44.3)

### Respondents' Awareness Regarding the Recommendations and Counseling for Prenatally-Diagnosed MCDK

All respondents reported giving initial information on the prognosis or treatment options. The majority of respondents (*n* = 99, 65.6%) counseled more than 12 families per year regarding prenatally-diagnosed MCDK. Eighty-two surgeons (54.3%) gave positive counseling regarding the baby's survival and future quality of life; 8/151 surgeons advised pregnancy termination; and 39.1% of the participating surgeons recommended genetic testing for pregnant women. Of the 8 respondents advising pregnancy termination, 3 (37.5%) were from Northeast China (the highest proportion of 8 respondents). Of the 59 respondents recommending genetic testing, 34 (57.6%) were from East China (the highest proportion of 59 respondents).

### Respondents' Knowledge, Attitudes, and Practices Regarding the Treatment and Natural History of MCDK

Regarding familiarity with MCDK, 31 (20.5%) respondents stated that they were not familiar with the literature. Ninety-nine surgeons (65.6%) were familiar with the literature and advocated the conservative management of MCDK. A small percentage of respondents stated that they were familiar with the literature (*n* = 21, 13.9%) but would suggest nephrectomy as an initial choice for patients with MCDK. Reasons for not using a “watch and wait” strategy included: not being familiar with the literature, parents' emotional distress, and concern about the risk of malignancy and hypertension. 70.4% of all respondents reported that initially suggesting nephrectomy is over-treatment.

### Practices of Surgeons Regarding the Choice of Imaging Modalities and Follow-Up for MCDK

A total of 33.1% of the sampled surgeons reported that routine VCUG should be performed in asymptomatic children with MCDK. Overall, 52% of surgeons indicated that they “almost always” perform renal scans to confirm the absence of function in the MCDK. Only 14.8% of respondents suggested limiting the number and frequency of radiographic evaluations for MCDK. More than half (51%) reported that renal ultrasonography should be performed after puberty.

### Attitudes of Surgeons Regarding a “Watch and Wait” Strategy for MCDK

For MCDK in children, a greater proportion of surgeons practicing for 11–20 years (77.3%) or >20 years (72.2%) selected a “watch and wait” strategy compared with those practicing <5 years (59%) or 5–10 years (58%). However, the differences were not significant (*P* = 0.08, Table 2). Surgeons working in academic teaching facilities were more likely to select a “watch and wait” strategy (81.7%) than those working in private practice (33.3%) and local or community hospitals (30%) (*P* < 0.001, Table 3). There was no difference between surgeons with higher education levels compared with surgeons with comparatively lower education levels regarding selecting a “watch and wait” strategy (*P* = 0.30, Table 4). Surgeons from East China had significantly higher referral rate of “watch and wait” strategy than those from other regions in China (*P* < 0.001, Table 5). Moreover, the recommendation rate of pediatric urologists for a “watch and wait” strategy are higher than that of doctors certified in pediatric surgery (*P* < 0.001, Table 6).

**Table 2:** Online comparison of respondents' attitudes to MCDK based on years of experience.

	**<5**	**5–10**	**10–20**	**>20**	***P***
**Prenatal diagnosed MCDK**					
Positive counseling no. (%)	21 (53.8)	20 (40)	25 (56.8)	16 (88.9)	0.02
Recommend termination of pregnancy No. (%)	2 (5.13)	3 (6)	2 (4.5)	1 (5.6)	0.95
Recommend genetic diagnosis No. (%)	14 (35.9)	20 (40)	16 (36.4)	9 (50)	0.50
**Children with MCDK**					
“Watch and wait” strategy No. (%)	23 (59)	29 (58)	34 (77.3)	13 (72.2)	0.08

*MCDK, multicystic dysplastic kidney*.

**Table 3:** Comparison of respondents' attitudes to MCDK based on practice setting.

	**Academic teaching facility**	**Public/government hospital**	**Local/community hospitals**	**Private practice**	***P***
**Prenatal diagnosed MCDK**					
Positive counseling No. (%)	47 (66.2)	29 (43.3)	4 (40)	2 (66.7)	0.03
Recommend termination of pregnancy No. (%)	1 (1.4)	2 (2.99)	4 (40)	1 (33.3)	<0.001
Recommend genetic diagnosis No. (%)	25 (35.2)	29 (43.3)	4 (40)	1 (33.3)	0.81
**Children with MCDK**					
“Watch and wait” strategy No. (%)	58 (81.7)	37 (55.2)	3 (30)	1 (33.3)	<0.001

*MCDK, multicystic dysplastic kidney*.

**Table 4:** Comparison of respondents' attitudes to MCDK based on education level.

	**Bachelor**	**Master and above**	***P***
**Prenatal diagnosed MCDK**			
Positive counseling No. (%)	15 (41.7)	67 (58.3)	0.08
Recommend termination of pregnancy No. (%)	3 (8.3)	5 (4.3)	0.61
Recommend genetic diagnosis No. (%)	19 (52.8)	40 (34.8)	0.05
**Children with MCDK**			
“Watch and wait” strategy No. (%)	21 (58.3)	78 (67.8)	0.30

*MCDK, multicystic dysplastic kidney*.

**Table 5:** Comparison of respondents' attitudes to MCDK based on location of practice.

	**Northeast**	**West China**	**East China**	**Central China**	***P***
**Prenatal diagnosed MCDK**					
Positive counseling No. (%)	11 (36.7)	13 (46.4)	50 (71.4)	8 (34.8)	0.001
Recommend termination of pregnancy No. (%)	3 (10)	2 (7.1)	1 (1.43)	2 (8.7)	0.11
Recommend genetic diagnosis No. (%)	6 (20)	11 (39.3)	34 (48.6)	8 (34.8)	0.06
**Children with MCDK**					
“Watch and wait” strategy No. (%)	9 (30)	16 (57.1)	60 (85.7)	14 (60.9)	<0.001

*MCDK, multicystic dysplastic kidney*.

**Table 6:** Comparison of respondents' attitudes to MCDK based on medical specialty.

	**Pediatric urology**	**Pediatric surgery**	***P***
**Prenatal diagnosed MCDK**			
Positive counseling No. (%)	49 (62.8)	33 (45.2)	0.03
Recommend termination of pregnancy No. (%)	2 (2.6)	6 (8.2)	0.12
Recommend genetic diagnosis No. (%)	23 (29.5)	36 (49.3)	0.01
**Children with MCDK**			
“Watch and wait” strategy No. (%)	61 (78.2)	38 (52.1)	<0.001

*MCDK, multicystic dysplastic kidney*.

## Discussion

With the increasing diagnosis of asymptomatic renal anomalies by comprehensive screening programs, MCDK has become one of the most commonly identified congenital anomalies of the urinary tract (13, 14). Now that the benign natural history of MCDK is more clearly understood, a non-surgical approach has recently become more popular (2, 4, 15). However, debate and problems have been continuing in the implementation process in China. Some Chinese scholars support surgical resection of the affected kidney because of concerns regarding the potential risks, such as malignancy and hypertension (12). In this report, we designed a survey to gather information about the opinions and practice patterns of pediatric urologists and pediatric surgeons from different parts of China regarding this question.

Our national survey found differences in the management practices used by pediatric urologists and pediatric surgeons from China, despite consensus regarding the importance of a “watch and wait” strategy for MCDK. Our results showed that the majority of participants were familiar with the literature discussing the management of MCDK. However, the use of a “watch and wait” strategy was not common among the Northeast China pediatric urologists and pediatric surgeons that we surveyed. Northeast is an underdeveloped inland region In China. The lack of education and training about knowledge of the management of MCDK among surgeons in this area may be a key factor in poor adherence to the suggested treatment.

Education level is the main factor affecting doctors' views on the diagnosis and management of some diseases (16, 17). However, education level was not associated with different attitudes toward the management of MCDK among pediatric urologists and pediatric surgeons in this study. This may indicate that surgeons with different education levels have the same opportunity to obtain new knowledge regarding MCDK in China. We also investigated respondents' attitudes to MCDK according to the practice setting. Surgeons from academic teaching facilities had higher referral rates for the “watch and wait” strategy than those from private and local/community hospitals. This result means that there are still imbalances between the attitudes of surgeons with respect to the management of MCDK. Practical experience may relatively weak in private and local/community hospitals in China. However, the number of respondents from private practice and local hospitals are very small. Therefore, the view that the imbalance of surgeons' knowledge and clinical practice in different levels of hospitals will lead to different perception of “watch and wait” strategy is still open to discussion.

This study showed that the recognition rate and actual recommendation rate of pediatric urologists for a “watch and wait” strategy are higher than that of doctors certified in pediatric surgery. Pediatric urologists specialize in congenital urinary system malformations. They have more opportunities to receive MCDK cases than pediatric surgeons. Therefore, pediatric urologists have more opportunities to try and verify new concepts and treatments for MCDK. The formation and change of doctors' perceptions may result from a combination of guideline recommendations, peer influence, and personal clinical practice experience.

Although there are now multiple opportunities for in-person or web-based learning for congenital diseases, our data show that doctors with longer working experience (>20 years) are significantly more likely to provide positive counseling for prenatally-diagnosed MCDK than other surgeons. This indicates that whether doctors provide positive counseling during prenatal consultations regarding the prognosis of MCDK depends on their accumulated clinical experience.

Patients' compliance with regular pregnancy examinations has increased, which provides a good opportunity for the early prenatal detection of MCDK. Recent studies described the genetic etiology of MCDK (13, 18–21), and many studies indicate that patients with MCDK are at increased risk for complications from other organic congenital abnormalities. These findings stress the need to explore the related gene mutation(s) in patients with MCDK. However, only 39.1% (59/151) of surgeons in our study recommended genetic testing when discussing prenatally-diagnosed MCDK. Previous studies also demonstrated that isolated MCKD often show a very low risk of chromosomal abnormality (13). Pediatric urologists and pediatric surgeons should pay more attention to prenatal genetic considerations in fetus MCDK with phenotypes of growth retardation or other organic congenital malformations.

Imaging examination plays an irreplaceable role during the follow-up of MCDK (22, 23). Ultrasonographic screening is not only of great significance in defining the development of MCDK, but also can prevent complications from infection or malignancy, thus, improving the quality of life for children with MCDK (23, 24). However, studies found that routine VCUG and renal nuclear medicine scans may not be warranted in children with MCDK without hydroureteronephrosis or signs and symptoms of urinary tract infections (9, 25). We also surveyed the attitudes of surgeons regarding imaging examinations for MCDK and found that 33.1% of the sampled surgeons in our study suggested performing VCUG in asymptomatic children. Moreover, only 14.8% of respondents suggested that the frequency of radiographic evaluation for MCDK be limited during follow-up. These results suggest that a large proportion of pediatric urologists and pediatric surgeons in China perform frequent imaging examinations during the follow-up of children with MCDK. According to the benign natural history of MCDK, frequent imaging examination has no clinical significance for the early detection of urinary infection, hypertension, or malignancy associated with MCDK (6, 14, 25–27). Therefore, Chinese pediatric urologists and pediatric surgeons should update their knowledge about the imaging frequency of follow-up in MCDK.

The main limitation in our study is the potential for sampling bias; most of the doctors surveyed in our study were from academic teaching facilities and public/government hospitals. Therefore, our survey results may be not reflect surgeons' knowledge level and treatment selection in other hospitals. Secondly, this study is not a representative survey because of only 151 responses from pediatric surgeons/urologist in China. Although the accuracy of our results will be greatly improved if data from all Chinese pediatric urologists and pediatric surgeons can be collected, it is important to work educatively now. We believe that “watch and wait” will be the new standard of the treatment of MCDK in China in a few years after increasing efforts is made to standardize clinical practice.

## Conclusion

In conclusion, this study aimed to evaluated surgeons' attitudes and practices regarding a “watch and wait” strategy for the treatment of MCDK. To our knowledge, ours is the first study to investigate the current perceptions and attitudes of Chinese pediatric urologists and pediatric surgeons regarding MCDK. We found that the knowledge level and acceptance of a “watch and wait” strategy for MCDK in these surgeons are still low. Expert consensus on a “watch and wait” strategy and standardized follow-up protocol are urgently needed in China to spread this strategy. The establishment of Chinese “watch and wait” registration database may provide more evidence for the use of this strategy. It will also guide and promote non-surgical treatment in clinical practice, so that more MCDK patients will directly benefit from non-surgical management. A larger sample size is required to fully identify the current opinions of Chinese pediatric urologists and pediatric surgeons regarding the management of MCDK.

## Data Availability Statement

All datasets generated for in this study are included in the article/supplementary material.

## Ethics Statement

The studies involving human participants were reviewed and approved by Shanghai Children's Medical Center Research Ethics Committee (SCMCEC–K2019040). The patients/participants provided their written informed consent to participate in this study.

## Author Contributions

DJ contributed to conception, design of the study, funding acquisition, and wrote the first draft of the manuscript. QW and ZS participated in the performance of the research, performed the statistical analysis, and wrote sections of the manuscript. All authors contributed to manuscript revision, read, and approved the submitted version.

## Conflict of Interest

The authors declare that the research was conducted in the absence of any commercial or financial relationships that could be construed as a potential conflict of interest.
